# User recommendation method integrating hierarchical graph attention network with multimodal knowledge graph

**DOI:** 10.3389/fnbot.2025.1587973

**Published:** 2025-06-18

**Authors:** Xiaofei Han, Xin Dou

**Affiliations:** ^1^Business College, California State University, Long Beach, CA, United States; ^2^School of Business and Management, Shanghai International Studies University, Shanghai, China

**Keywords:** user recommendation, hierarchical graph attention network, knowledge graph, multimodal, visual features, textual features

## Abstract

In common graph neural network (GNN), although incorporating social network information effectively utilizes interactions between users, it often overlooks the deeper semantic relationships between items and fails to integrate visual and textual feature information. This limitation can restrict the diversity and accuracy of recommendation results. To address this, the present study combines knowledge graph, GNN, and multimodal information to enhance feature representations of both users and items. The inclusion of knowledge graph not only provides a better understanding of the underlying logic behind user interests and preferences but also aids in addressing the cold-start problem for new users and items. Moreover, in improving recommendation accuracy, visual and textual features of items are incorporated as supplementary information. Therefore, a user recommendation model is proposed that integrates hierarchical graph attention network with multimodal knowledge graph. The model consists of four key components: a collaborative knowledge graph neural layer, an image feature extraction layer, a text feature extraction layer, and a prediction layer. The first three layers extract user and item features, and the recommendation is completed in the prediction layer. Experimental results based on two public datasets demonstrate that the proposed model significantly outperforms existing recommendation methods in terms of recommendation performance.

## 1 Introduction

In the era of rapid information expansion, users are increasingly overwhelmed by vast amounts of digital content, leading to issues such as information overload and difficulty in efficiently locating relevant content. This challenge is particularly pronounced on emerging short video, social media, and e-commerce platforms, where the volume and diversity of data far exceed users’ cognitive and processing capacities. Consequently, intelligent recommendation systems have become essential tools for filtering massive datasets and delivering personalized content to users ((Liu et al., 2024; Wu et al., 2024; Yamada, 2021) and limited representation capacity, all of which hinder their performance in dynamic and complex environments (Zhang et al., 2019; Gao et al., 2022). With the rise of deep learning, the landscape of recommendation systems has evolved significantly. Techniques such as convolutional neural network (CNN) and recurrent neural networks (RNN) have shown strong capabilities in feature extraction and modeling temporal user preferences (Han et al., 2024; Wang et al., 2024, 2025, Yan et al., 2025).

Traditional recommendation approaches—such as content-based filtering (CBF), collaborative filtering (CF), and hybrid recommendation (HR)—primarily rely on users’ historical interactions (e.g., purchases or browsing records) and similarity computations to provide recommendations (Javed et al., 2021; Wu et al., 2023). While these methods have achieved moderate success, they struggle with key issues such as the cold start problem, data sparsity, and limited representation capacity, all of which hinder their performance in dynamic and complex environments (Zhang et al., 2019).

With the rise of deep learning, the landscape of recommendation systems has evolved significantly. Techniques such as convolutional neural network (CNN) and recurrent neural networks (RNN) have shown strong capabilities in feature extraction and modeling temporal user preferences (Steck et al., 2021; Bandyopadhyay et al., 2024). For example, Visa et al. proposed a CNN-based feature extraction method, which uses matrix multiplication between users and items to uncover latent relationships, effectively solving the sparsity issue of similarity matrices and optimizing recommendation results (Visa and Patel, 2021). Cho et al. designed an RNN-based recommendation system that analyzes temporal data to precisely capture dynamically changing user needs, providing more accurate recommendations (Cho et al., 2014). More recently, graph neural networks (GNNs) have demonstrated remarkable effectiveness in capturing intricate relationships between users and items by modeling them as graph structures (Jiang et al., 2023). For instance, the GraphRec model proposed by Fan et al. constructs a user information model that combines social network data with user characteristics and uses a multilayer perceptron to extract features of target items, improving recommendation accuracy (Fan et al., 2019). Chen et al. improved the recommendation system’s efficiency by leveraging social neighbor network information and using heterogeneous GNN methods (Chen and Wong, 2021).

Parallel to these advances, knowledge graphs (KG) have emerged as a promising auxiliary resource for enhancing recommendation performance. By formally encoding entities and their semantic relationships, KGs enable richer user-item interaction modeling. Existing KG-enhanced recommendation strategies include embedding-based, path-based, and joint learning approaches. These techniques have demonstrated improved interpretability and accuracy by incorporating external structured knowledge into recommendation pipelines (Guo et al., 2020; Zhang et al., 2024). Oramas et al. (2016) proposed a recommendation system that combines semantic and collaborative characteristics by extracting information from KG using both entity-and path-based strategies, transforming it into linear features. Path-based recommendations involve establishing user-item relation graph to uncover connections between entities, measure node similarity, and recommend content. Path analysis in KG reveals complex relationships between entities, allowing for precise exploration of user preferences through specific meta-paths, enhancing the interpretability of the recommendation system (Sun et al., 2020). Ma et al. (2019) developed the RuleRec algorithm, which extracts rules from an item-centric KG to identify various associations and provides recommendations using these inferred rules. Joint recommendation methods integrate path analysis and knowledge graph embedding approaches, where user interests are first captured via a propagation mechanism across the entire knowledge graph and then extracted through graph embedding techniques, ultimately completing the recommendation process (Yang et al., 2022). Wang X. et al. (2019) proposed the model, which combines user-item bipartite graph with knowledge graph and performs iterative diffusion in shared knowledge graph via GNN, enriching entity embeddings. Shi et al. (2021) introduced the method, modeling a heterogeneous information network, extracting multi-dimensional similarity matrices using different meta-paths, and integrating this information through deep learning network to complete the recommendation process.

Furthermore, multimodal recommendation systems—which integrate visual, textual, and sometimes audio data—have received growing attention for their ability to address the limitations of single-modal systems and further refine personalization (Li et al., 2019). By leveraging the complementary nature of different data types, multimodal methods can offer more comprehensive user representations and deeper insights into user preferences.

Motivated by the limitations of traditional and single-modal recommendation methods, this study proposes a novel recommendation framework that integrates hierarchical graph attention networks with a multimodal knowledge graph (HGAN-MKG). The model consists of four major components: a collaborative knowledge graph neural layer, an image feature extraction layer, a text feature extraction layer, and a prediction layer. Specifically, the collaborative KG layer captures deep user-item interactions via attention mechanisms and gated recurrent unit (GRU); the image layer applies a multi-path attention structure to analyze visual user behavior; the text layer uses multi-head self-attention and CNN to extract contextual features; and the prediction layer fuses all modalities to generate accurate recommendations. Experiments conducted on two benchmark datasets confirm the effectiveness of the proposed model, which outperforms several state-of-the-art baselines.

The remainder of this study is organized as follows: Section 2 introduces the underlying theory, Section 3 describes the design details of the algorithm, Section 4 presents experimental validation, and Section 5 concludes the research work.

## 2 Theoretical foundations

### 2.1 K-means clustering algorithm

The K-Means algorithm is an effective data clustering method that partitions a dataset into *K* clusters, with each cluster associated with its nearest center (Bock, 2007). This iterative algorithm continuously updates cluster centers and reassigns points to clusters until a predefined stopping criterion is met. The algorithm’s principle is determined by solving an optimization function, with the sum of squared errors serving as the evaluation metric. The objective is to minimize the total cost function, as defined in Equation 1.

(1) $$T=\sum_{m=1}^{K}\sum_{n=1}^{K}(x_{n}-\partial_{m})$$

where $\partial_{m}$ represents the centroid of cluster 𝑚, 𝑥_𝑛_ is an element from the sample set, and 𝐾 denotes the number of clusters.

In the context of recommendation systems, *K*-Means is applied to group users based on behavior, converting user activity data into vector form, and clustering users into 𝐾 groups. This method identifies similarities between users, enabling personalized recommendations for items that users within the same group are likely to find interesting.

### 2.2 Attention mechanism technologies

#### 2.2.1 Attention mechanism

**Figure 1:**
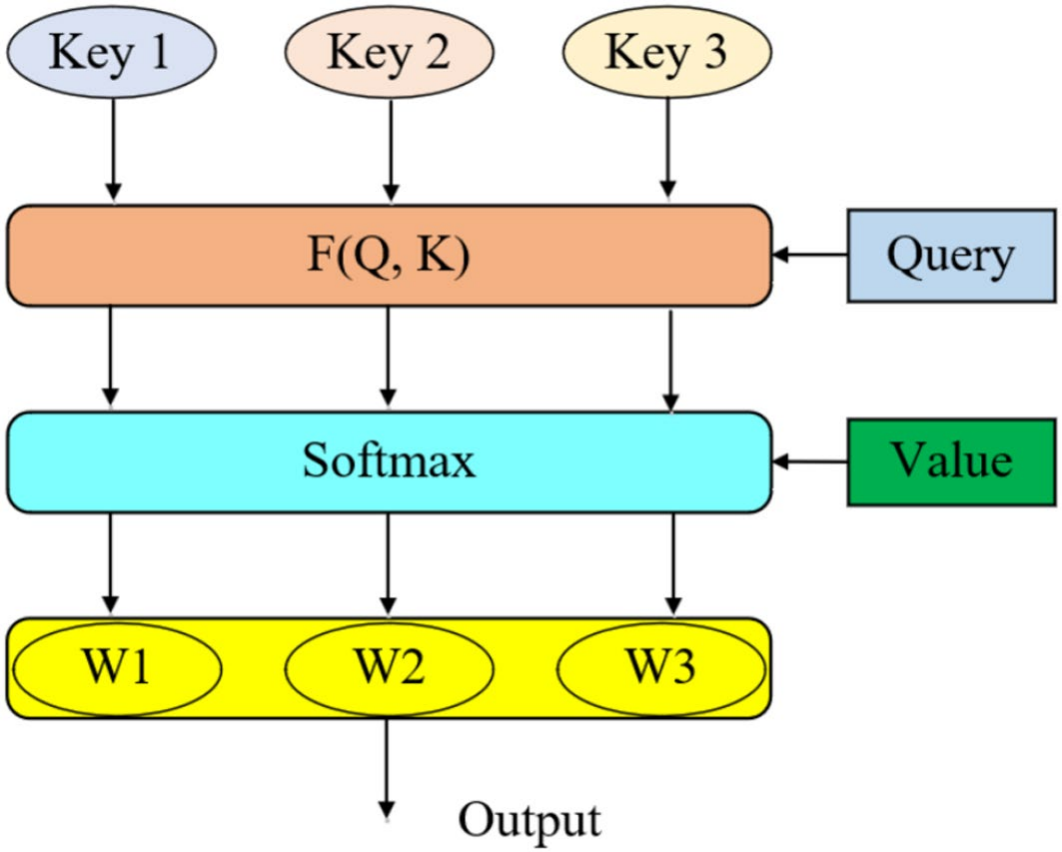
Allocation of attention mechanism weight.

The attention mechanism allocates different weights to various inputs based on their importance, prioritizing more relevant information while ignoring less significant details. As depicted in Figure 1, each input’s Key, Value, and Query are processed as vectors, and the weight for each Key is determined by calculating its similarity to the Query. This results in a weighted sum of all Values, generating the final output. The calculation of similarity can be approached through four distinct strategies which is presented in Equation 2.

(2) $$\begin{array}{c} F(Q,K_{i})=Q^{T}{\mathrm{WK}}_{i}\\ F(Q,K_{i})=\tanh(W[Q^{T};K_{i}])\\ F(Q,K_{i})=V^{T}\tanh(\mathrm{WQ}+{\mathrm{UK}}_{i})\\ F(Q,K_{i})=Q^{T}K_{i} \end{array}$$

#### 2.2.2 Graph attention mechanism

With the expansion of research in this field, GAN and their variants have emerged, becoming integral to GNN These mechanisms typically form the core of complex deep learning architectures involving multiple convolutional or pooling layers designed to handle graph-structured data. Each layer participates in the propagation and aggregation of features between nodes and their neighbors, updating node representations and performing classification tasks. The graph attention mechanism emphasizes the dynamic evaluation of relative importance between nodes, calculating weights accordingly. As a critical component of GNN, it plays a pivotal role in computing node weights and handling diverse graph structures and tasks.

This mechanism assigns weights based on input features, allowing for weighted aggregation of data for more precise and effective representation. By assessing similarities between nodes and their neighbors, attention coefficients are computed to allocate weights. This strategy enhances the significance of key nodes while mitigating noise interference. Typically, a trainable network model is used to determine attention coefficients, considering the unique features of nodes and their relative positions with neighboring nodes, ultimately generating attention weights. Once these weights are obtained, node feature vectors are combined with their corresponding weights, resulting in a weighted feature vector that represents either a node or the entire graph.

#### 2.2.3 Multi-head self-attention mechanism

The multi-head self-attention mechanism, a critical component of the Transformer model, has been widely adopted across various domains. Within the Transformer architecture, the attention mechanism consists of two key parts: scaled dot-product attention and multi-head attention, which together form the foundation of the model. The computation of scaled dot-product attention is as follows, as defined in Equation 3:

(3) $$\text{Attention}(Q,K,V)=\text{Softmax}(\frac{QK^{T}}{\sqrt{d_{k}}})V$$

where *Q* represents the query vector, *K* and *V* are the key-value pairs, and *d_k_* is the scaling factor.

Both single-head and multi-head self-attention are considered derivative forms of the attention mechanism, with multi-head attention enhancing the model’s ability to manage long sequences and their complexity. Given an input sequence [𝑤_1_, 𝑤_2_, 𝑤_3_, …, 𝑤_𝑇_], where each 𝑤_𝑖_ represents the vector form of the $i_{\mathrm{th}}$ word in the sequence.

**Figure 2:**
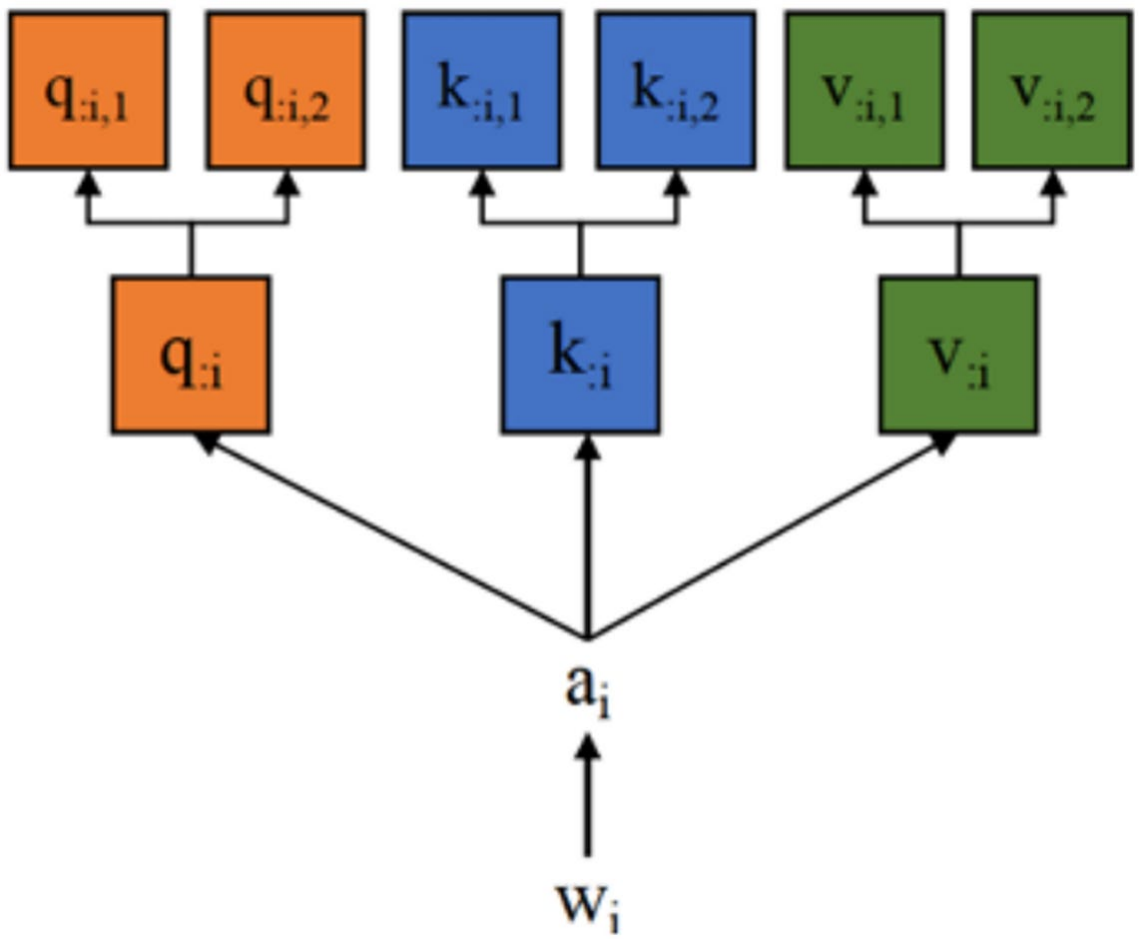
Multi-head self-attention.

The final representation 𝑏=[𝑏_1_, 𝑏_2_, 𝑏_3_, …, 𝑏_𝑟_] is derived from the weighted aggregation of attention heads. In a single-head attention mechanism, 𝑞_1_, 𝑘_1_, and 𝑣_1_ constitute one “head.” The multi-head self-attention mechanism multiplies specific 𝑤_1_ values with multiple 𝑊^𝑄^, 𝑊^𝐾^, and 𝑊^𝑉^ matrices to generate multiple sets of 𝑞_1_, 𝑘_1_, and 𝑣_1_, which is presented in Figure 2.

After obtaining the outputs from all heads, these feature vectors are concatenated and linearly transformed to generate the final representation as defined in Equation 4:

(4) $$\begin{array}{c} \text{Multihead}(Q,K,V)=\text{Concat}(\mathrm{hea}d_{1},\cdots ,{\text{head}}_{x})W^{O}\\ {\text{head}}_{i}=\text{Attention}(QW_{i}^{Q},KW_{i}^{K},VW_{i}^{V}) \end{array}$$

### 2.3 Knowledge graph

A knowledge graph is essentially a semantic structure represented in the form of a graph, containing multiple categories of entities and offering a more intuitive and transparent visualization of complex relationships. It effectively encodes semantic information between entities and provides a highly structured means of representation. Mapping the entities and their relations into a low-dimensional continuous vector space is a critical step toward knowledge modeling and enhancing recommendation systems. During this process, it is essential to preserve both the structural properties of the graph and the semantic consistency of the nodes, in order to minimize information loss or distortion.

Various techniques have been proposed for feature extraction in knowledge base construction, including distance-based embedding models, similarity-based conceptual models, and path-based relational learning methods. Among them, translational models have gained widespread adoption due to their simplicity and scalability. Representative distance-based translational models include TransE, TransR, and TransD. For instance, TransR models entities as collections of multi-attribute information and achieves triple-level embedding by projecting entities into relation-specific spaces.

In TransR, each relation *r* is associated with a distinct relation space, and a projection matrix $M_{r}$ is defined to map entity vectors from the entity space to the corresponding relation space. Given a head entity *h*, a tail entity *t*, and a relation *r*, their representations in the relation space are represented in Equation 5:

(5) $$\begin{array}{l} h_{⊥}=M_{r}h\\ t_{⊥}=M_{r}t \end{array}$$

where $h,t\in {\mathbb{R}}^{k}$ represents the vector representation of the entity in the original entity space, $M_{r}\in {\mathbb{R}}^{d\times k}$ is the projection matrix corresponding to the relation *r*, and $h_{⊥},t_{⊥}\in {\mathbb{R}}^{d}$ means the embedding representation of the head entity and the tail entity in the relation space. Through the above mapping, TransR can better model the differential impact of different relations on entity semantics.

**Figure 3:**
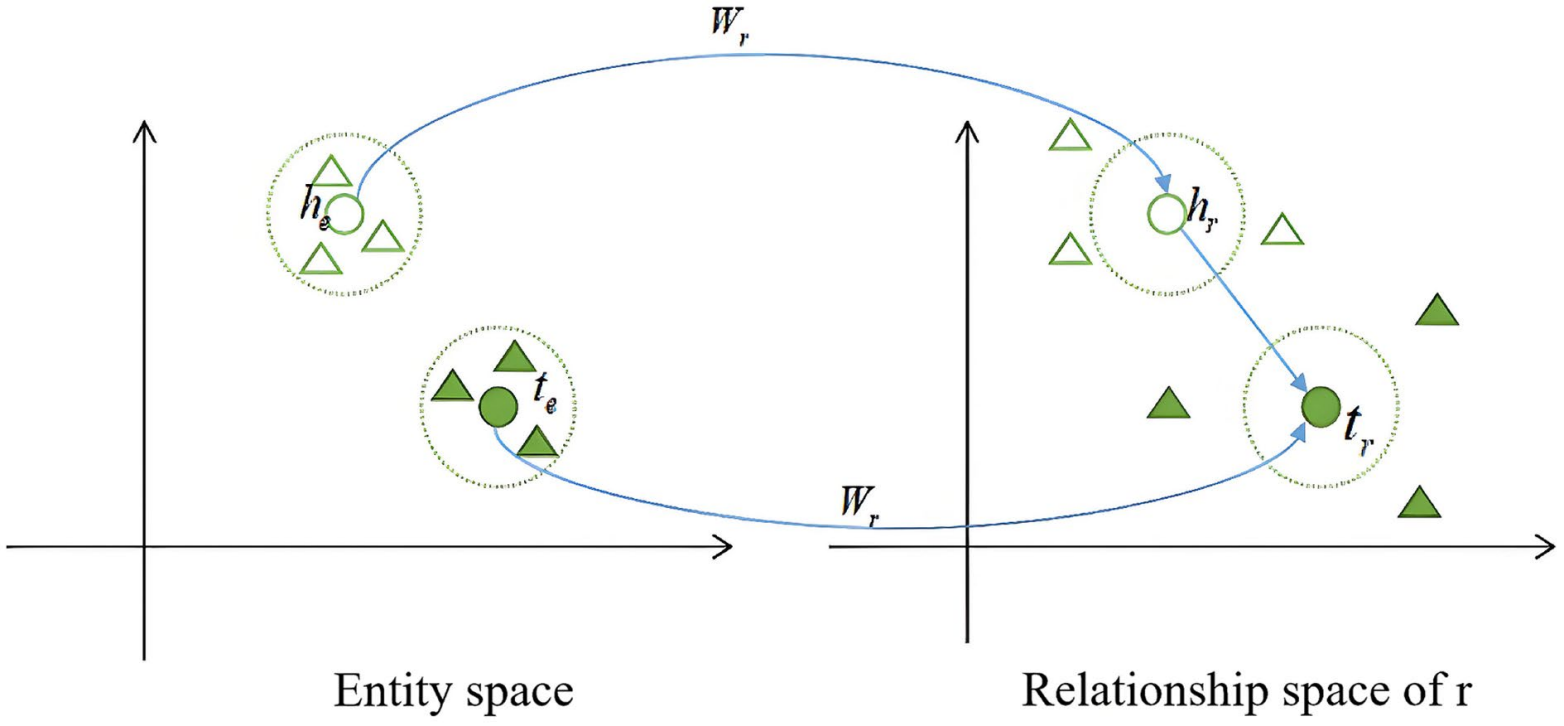
Graphical description of TransR.

Figure 3 illustrates the core concept of TransR. For each triple (ℎ+𝑟), the entities ℎ and 𝑡 are mapped to the relationship space *r* through a projection matrix $W_{r}$ , resulting in representations ℎ_𝑟_ and 𝑡_𝑟_. The goal is to ensure that (ℎ_𝑟_+𝑟) closely approximates 𝑡_r_. The transformation of entities in the space is represented by the following Equation 6:

(6) $$h_{r}={\mathrm{hW}}_{r},t_{r}={\mathrm{tW}}_{r}$$

The score function for determining the proximity between head and tail entities is given by Equation 7:

(7) $$g(h,r,t)=h_{r}+r-{t_{r}}_{2}^{2}$$

According to Equation 7, the lower the score of the triple, the closer the head and tail entities are in the relationship space 𝑟, which increases the probability of the triple being correct.

## 3 Methodology

**Figure 4:**
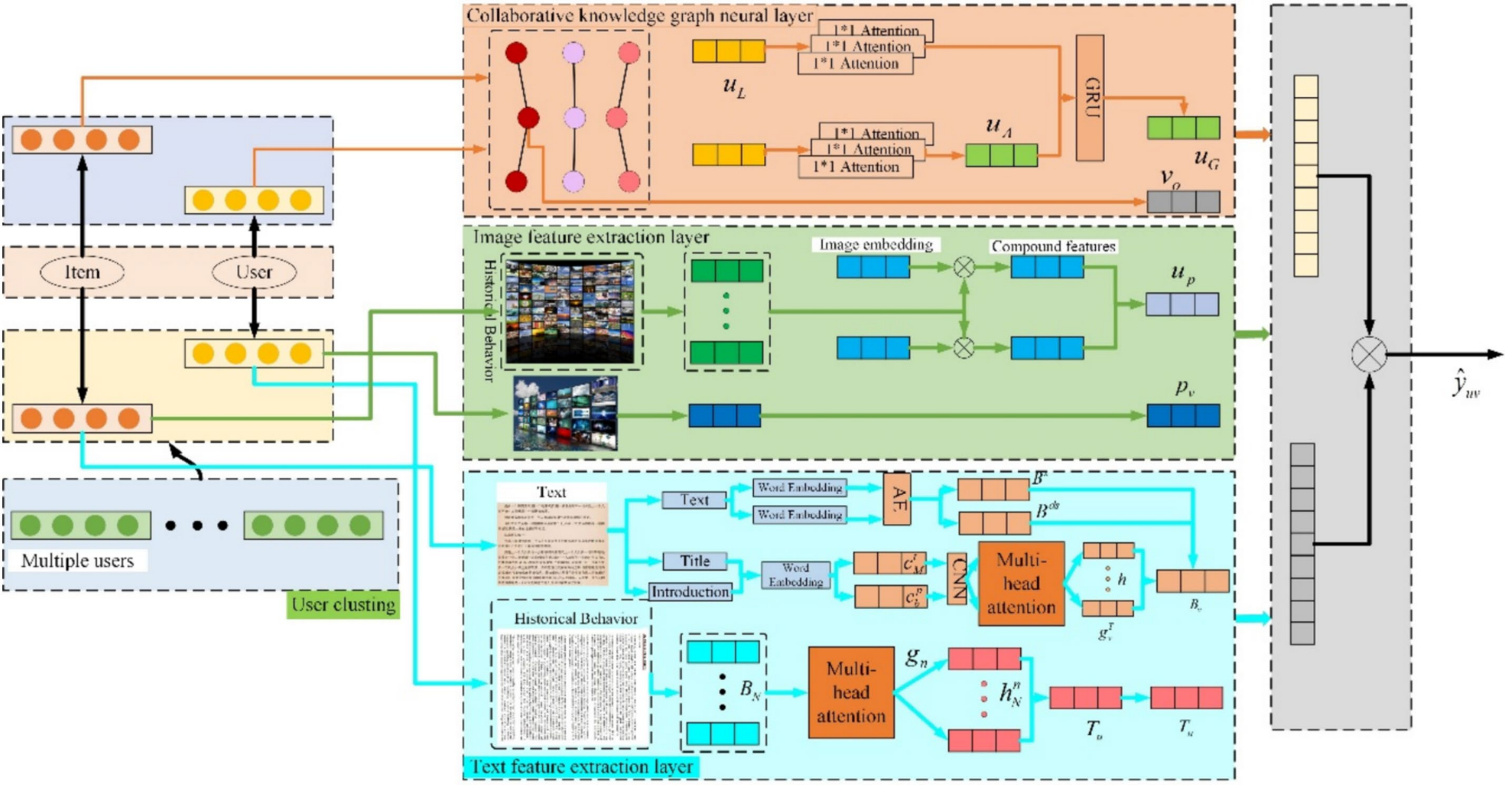
User recommendation method integrating hierarchical graph attention network with multimodal knowledge graph.

The proposed recommended model HGAN-MKG, first extracts knowledge graph information. It then utilizes the VGG19 network to extract image features of items and combines multi-head self-attention mechanisms with CNN to extract text features. Finally, the model fuses these three types of information for recommendation purposes. The proposed model consists of four key components: the collaborative knowledge graph neural layer, the image feature extraction layer, the text feature extraction layer, and the prediction network layer. The model architecture is illustrated in Figure 4.

### 3.1 Collaborative knowledge graph neural layer

In the collaborative knowledge graph neural layer, the bipartite graph between users and items in the knowledge graph is integrated to connect item features, thereby forming a knowledge graph. Let 𝑉={𝑣_1_, 𝑣_2_, …, 𝑣_𝑚_} represent the set of items and 𝑈={𝑢_1_, 𝑢_2_, …, 𝑢_𝑛_} represent the users. The set 𝐸={𝑒_1_, 𝑒_2_, …, 𝑒_𝑜_} corresponds to the set of entities, where 𝑜, 𝑛, and 𝑚 denote the total number of entities, users, and items, respectively. The matrix 𝑌_𝑖𝑗_ indicates the interactions between users and items, defined as 𝑌_𝑖𝑗_ =1 if an interaction exists, and 𝑌_𝑖𝑗_=0 otherwise. A knowledge graph 𝐺={(ℎ, 𝑟, 𝑡)|ℎ, 𝑟=𝜀, 𝑟∈𝑅} is defined, where each triple consists of a head entity ℎ, a relationship 𝑟, and a tail entity 𝑡. If there is an association between ℎ and 𝑡, the elements in GGG represent entities and their associations.

In practice, the representation of entities and their relationships within a KG is typically approached using translation-based learning methods. These methods perform logical reasoning and mapping of entities and their relationships in a low-dimensional space for knowledge representation. In the TransE model, the triple (ℎ, 𝑟, 𝑡) is expressed in vectorized form, where the relationship 𝑟 is understood as a translation from ℎ to 𝑡. By fine-tuning the vector representations of the triples, the equation ℎ+𝑟 ≈𝑡 is satisfied. As technology has advanced, derivative techniques have emerged, such as the TransR model, where each entity is viewed as having multiple facets. Different relationships correspond to different aspects of an entity, and each semantic space corresponds to a specific relationship.

Through embedding techniques, item vector representations are obtained, and the attention mechanism is applied to explore the interactions between entities within the knowledge graph. This method not only integrates user data but also incorporates the internal relationships and complex hierarchical structures between entities in the knowledge graph. The training process based on the attention mechanism involves random sampling of nodes and the calculation of weight scores to capture direct relationships between entities. Subsequently, the attention mechanism analyzes these weights to identify and understand first-order entity relationships, which can be further extended to 𝐿-order entity relationships.

This paper delves into the training methods for the initial stages and extends the analysis to multi-layer 𝐿-structures. In this network layer, Equation 8 reveals the interactions between two entities, while Equation 9 measures the degree to which a user prefers specific relationships and entity information. For a specific item 𝑣_0_, its neighboring nodes in the set $v_{U_{v^{0}}}^{u}$ are described by the formula, where (𝑟, 𝑖, 𝑗) defines the connections between entities, and the attention mechanism is used to compute and evaluate the weights.

(8) $$e_{j}r_{i,j}=\sigma (W_{1}\text{gconcat}(r_{i,j},e_{j})+b_{1})$$

(9) $$\pi_{r_{i},e_{j}}^{u}=u\cdot r_{i,j}e_{j}$$

where 𝑢∈R, 𝑟_𝑖,𝑗_ ∈R, j∈R, and 𝑒_𝑗_ ∈R represent the user, entity connections, and tail entity vector representations, respectively, with www denoting the dimensionality. The structure uses a non-linear activation function *σ* and adjusts the weights 𝑊_1_ and bias 𝑏_1_∈𝑅 to assess the strength of user interest in different relationships and entity data. To construct the vectorized representation of neighboring entities for item 𝑣_0_, the vectors of adjacent entities 𝑒_0_ are linearly fused as shown in Equation 10, followed by subsequent normalization.

(10) $$\begin{array}{l} v_{U_{v^{0}}}^{u}=\sum_{e^{0}\in U_{v^{0}}}{\bar{\pi }}_{r_{i,j},e_{j}}^{u}e^{0}\\ {\bar{\pi }}_{r_{i,j},e_{j}}^{u}=\frac{\exp(\pi_{r_{i,j},e_{j}}^{u})}{\sum_{e^{0}\in U_{v^{0}}}{\bar{\pi }}_{r_{i,j},e_{j}}^{u}e^{0}} \end{array}$$

To explore deeper entity information, this layer is extended from a single level to multiple layers, resulting in 𝐿-order vectorized descriptions of entities. This 𝐿 -order vector representation aggregates data from neighboring entities up to (𝐿−1)-order. The final vector representation of the item 𝑣_𝐿_ is obtained, where 𝑊_𝐿_ represents the weight parameters and 𝑏_𝐿_ denotes the bias. This is calculated precisely using Equation 11.

(11) $$v_{L}=\sigma (W_{L}\cdot (v_{L-1}+v_{U_{v}L})+b_{L})$$

Ultimately, a summary analysis is conducted to derive a representation 𝑢_A_, reflecting the user’s short-term interests, which is illustrated in Equation 12:

(12) $$u_{A}=\sum_{i=1}^{N}\alpha_{i}v_{u,i}$$

where 𝑣_𝑢,𝑖_ represents the user’s *N*-order preference {𝑣_𝑢,1_, 𝑣_𝑢,2_,…𝑣_𝑢,𝑁_}, and 𝛼_𝑖_ represents the attention weight coefficients. These coefficients are calculated based on the spatial relationships between entities in the knowledge graph as illustrated in Equation 13:

(13) $$\alpha_{i}=\frac{\exp(h,r,t)}{\sum_{i=1}^{N}\exp(h,r,t)}$$

To more accurately capture the user’s final interests, vectors generated by the embedding layer are used to represent the user’s long-term interests. At the same time, aggregated vectors processed by the attention mechanism reflect the user’s short-term interests. A GRU model is then applied to integrate both long-term and short-term interests, forming a comprehensive representation of the user’s interest preferences. GRU is adopted due to its efficient gating mechanism and relatively lower computational complexity compared to LSTM, while maintaining comparable performance in modeling sequential dependencies. Unlike Transformer-based models, which typically require large-scale training data and extensive tuning, GRU provides a lightweight and effective solution for learning user preferences in data-constrained or latency-sensitive scenarios. The initial set of items interacting with the user is denoted as 𝑣_𝑜_.

User long-term preference representations 𝑢_𝐿_ are trained using historical interaction data. These are then combined with short-term preferences 𝑢_𝐴_ and trained through the GRU model. After training, the selected hidden layer undergoes normalization to produce the final representation of the user’s preference 𝑢_𝐺_. Ultimately, the long-term preferences 𝑢_𝐿_ are fused with the short-term preferences 𝑢_𝐴_, and a deep GRU model is used for training. The resulting hidden layers are normalized to generate the final user preference expression 𝑢_𝐺_ as shown in Equation 14:

(14) $$u_{G}=\sigma (W_{4}[u_{L},u_{A}]+b_{4})$$

### 3.2 Image feature extraction layer

In the image feature extraction layer, K-Means clustering is first applied to the image features corresponding to the user’s interaction history, aiming to uncover latent user preference patterns. Specifically, image features are extracted using a pretrained VGG19 model (employing its static components from Conv1 to FC7 layers), and uniformly transformed into 4,096-dimensional vector representations. These feature vectors are then input into a K-Means clustering algorithm to generate a set of representative cluster centroids, each corresponding to a latent category of visual preference. The resulting clusters are utilized not only for modeling user interests in visual content but also as input for subsequent dynamic feature learning. To further capture semantic representations of these clustered features at the individual user level, a trainable feature modeling module composed of three fully connected layers is constructed. This module is designed to generate high-level semantic representations of images. The computational process is defined as follows, as shown in Equation 15:

(15) $$\begin{array}{c} s_{0}=\text{Tanh}(W_{a}p_{o}+b_{a})\\ p_{v}=W_{c}P\cdot \text{ReLU}(W_{b}s_{0}+b_{b})+b_{c} \end{array}$$

where $p_{o}\in {\mathbb{R}}^{4096}$ denote the initial image feature vector extracted via VGG19, and $s_{0}$ the intermediate semantic representation. The matrices $W_{a}$ , $W_{b}$ , and $W_{c}$ , along with the corresponding biases $b_{a}$ , $b_{b}$ , and $b_{c}$ , represent the weights and biases of the fully connected layers, respectively. The input *p* refers to the feature vector obtained after clustering, and $p_{v}$ denotes the final semantic representation vector of the image.

**Figure 5:**
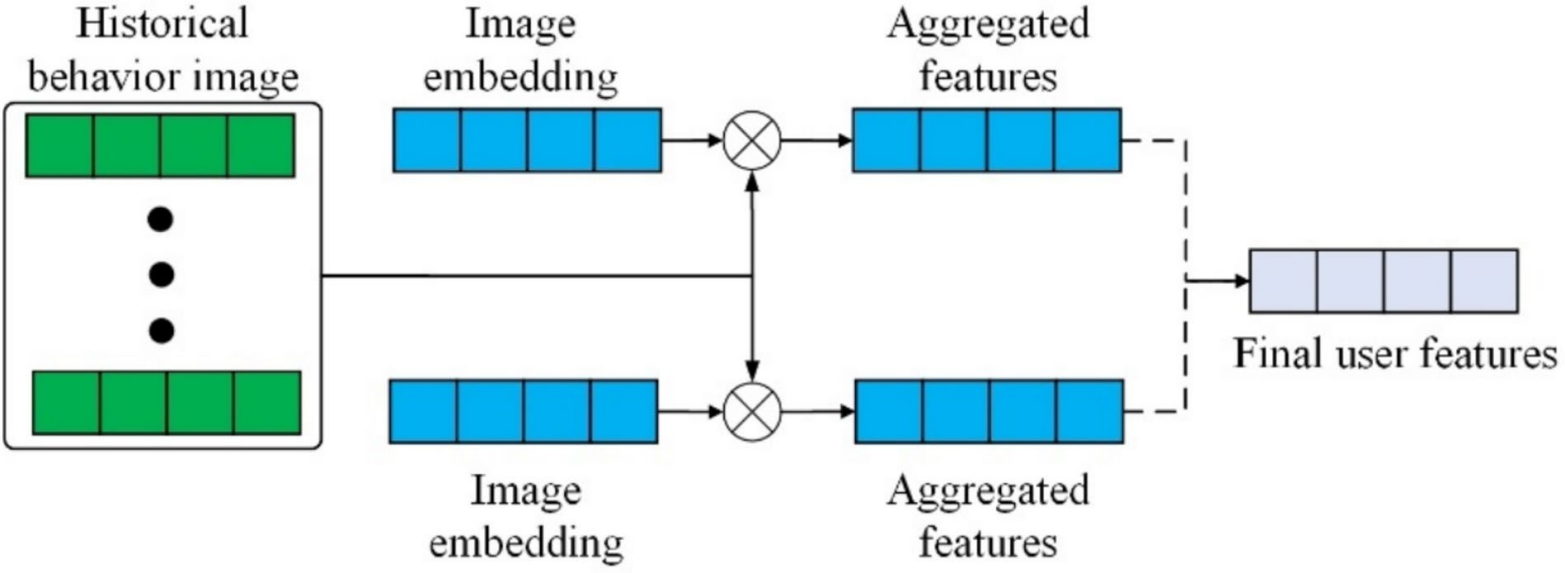
Structure of image feature aggregation.

In this section, the first 18 layers of the VGG19 network are fixed as output 𝑝_𝑜_, which is used to extract the visual features 𝑝_𝑣_ of items. The images representing user-item interactions capture the user’s visual preferences. Since the contribution of these historical interaction images to capturing user preferences varies, an image aggregation network is employed to integrate these images differentially. The specific structure of the aggregation network is shown in Figure 5. The network utilizes a multi-channel attention mechanism, where the item’s image features and the initial representation of the target item serve as query vectors. These vectors are then passed through various fully connected networks to form individual weights and weighted vectors. The weighted vectors from the two channels are then aggregated to obtain a visual representation of the user’s historical behavior, calculated as shown in Equation 16:

(16) $$u_{p}=\varphi p_{u}^{1}+(1-\varphi )p_{u}^{2}$$

This represents the set 𝑝_𝑖_ of images from items previously interacted with by the user. The image of the $i_{\mathrm{th}}$ item is transformed into a vector through embedding technology, which also includes the embedded representation of the target item image 𝑝_𝑣_. The training parameters are denoted by 𝜑, and the formulas for $p_{u}^{1}$ and $p_{u}^{2}$ are as shown in Equation 17:

(17) $$\begin{array}{l} p_{u}^{1}=\sum_{i=1}^{N}g_{p}(p_{i},p_{v})p_{i}\\ p_{u}^{2}=\sum_{i=1}^{N}g_{p}(p_{i},p_{o})p_{i} \end{array}$$

### 3.3 Text feature extraction layer

This layer is responsible for extracting textual features. K-Means clustering is first applied to the user data before proceeding with further operations. For text feature extraction, consider a movie as an example. A movie’s textual features include its title, genre, and description, all of which are logically related. In this network layer, multi-head self-attention is employed to extract features from each component (movie title, genre, and description), which are then fused to form the final textual feature representation.

Regarding item categories, using movies as an example, when a user selects a movie for viewing in an app, they generally choose a broad genre, such as comedy, romance, or drama. If the user selects a comedy, they may further choose from sub-genres like slapstick, satirical, dark humor, or martial arts comedy. Similar patterns apply to other genres. Therefore, for category features, both broad and fine-grained categories are considered for feature extraction. During embedding, the ID is input and transformed into low-dimensional representations 𝑒^𝑠^ and 𝑒^𝑑𝑠^. The hidden category representation is then learned as shown in Equation 18:

(18) $$\begin{array}{c} B^{s}=\text{ReLU}(V_{s}\times e^{s}+v_{s})\\ B^{\mathrm{ds}}=\text{ReLU}(V_{\mathrm{ds}}\times e^{\mathrm{ds}}+v_{\mathrm{ds}}) \end{array}$$

Next, the item title is used to obtain its feature representation. This part involves three steps. First, the word sequence of the item title is transformed into a low-dimensional semantic vector sequence, converting the sequence of title words $\left[w_{1}^{t},w_{2}^{t},\cdots ,w_{M}^{t}\right]$ into a vector $\left[c_{1}^{t},c_{2}^{t},\cdots ,c_{M}^{t}\right]$ . The CNN component then extracts short-range contextual features from the words in the movie title. Using CNN, the context representation of the $i_{\mathrm{th}}$ word is derived as $c_{i}^{t}$ , calculated as shown in Equation 19:

(19) $$e_{i}^{t}=\text{ReLU}(F_{t}\times c_{\left(i-X):(i+X\right)}^{t}+b_{t})$$

where $c_{\left(i-X):(i+X\right)}^{t}$ represents the concatenation of word embeddings from positions (𝑖−𝑋) to (𝑖+𝑋). $F_{t}$ and $b_{t}$ are the kernel and bias parameters of the convolutional neural network. The CNN output sequence is $\left[e_{1}^{t},e_{2}^{t},\cdots ,e_{M}^{t}\right]$ . A similar approach is applied to the movie description sequence.

The final step uses multi-head self-attention to model the relationships between components, enabling better capture of distant textual features. The $k_{\mathrm{th}}$ attention head’s representation of the $i_{\mathrm{th}}$ word is calculated as shown in Equation 20:

(20) $$\begin{array}{l} a_{i,j}^{k}=\frac{\exp(e_{i}Q_{k}^{w}e_{j})}{\sum_{m=1}^{M}\exp(e_{i}Q_{k}^{w}e_{m})}\\ h_{i,j}^{k}=V_{k}^{w}(\sum_{j=1}^{M}a_{i,j}^{k}e_{j}) \end{array}$$

where $Q_{k}^{w}$ and $V_{k}^{w}$ are the projection parameters of the self-attention head, and $a_{i,j}^{k}$ represents the relative importance of interactions between the $i_{\mathrm{th}}$ and $j_{\mathrm{th}}$ words. The multi-head representation $h_{i}^{w}$ for the $i_{\mathrm{th}}$ word is obtained by concatenating the representations from the *h* independent attention heads.

Since the same word carries varying amounts of critical information across different item components, attention is used to assign weight proportions. The calculation for this is as shown in Equation 21:

(21) $$a_{i}^{w}=\frac{\exp(a_{i}^{w})}{\sum_{j=1}^{M}\exp(a_{j}^{w})}$$

The final representation of each component is obtained by aggregating the weighted expressions of the words. The calculation is as shown in Equation 22:

(22) $$B=\sum_{i=1}^{M}a_{i}^{w}h_{i}^{w}$$

Given that different components contain varying amounts of information—titles and descriptions may carry more relevant details, while categories more accurately represent the item’s attributes—attention is employed to balance the weights, reflecting the amount of information each component carries. The calculation is as shown in Equation 23:

(23) $$\begin{array}{l} a_{\mathrm{tb}}=g_{v}^{T}\tanh(U_{v}^{h}\times B^{\mathrm{tb}}+u_{v})\\ a_{\mathrm{tb}}=\frac{\exp(a_{\mathrm{tb}})}{\exp(a_{\mathrm{sc}})+\exp(a_{c})+\exp(a_{\mathrm{tb}})} \end{array}$$

Similarly, the attention weights for categories and sub-categories are denoted as 𝑎_𝑠_ and 𝑎_𝑑𝑠_. The final representation is the weighted sum of the component representations, as presented in Equation 24:

(24) $$B=a_{\mathrm{tb}}B^{\mathrm{tb}}+a_{\mathrm{ds}}B^{\mathrm{ds}}+a_{s}B^{s}$$

**Figure 6:**
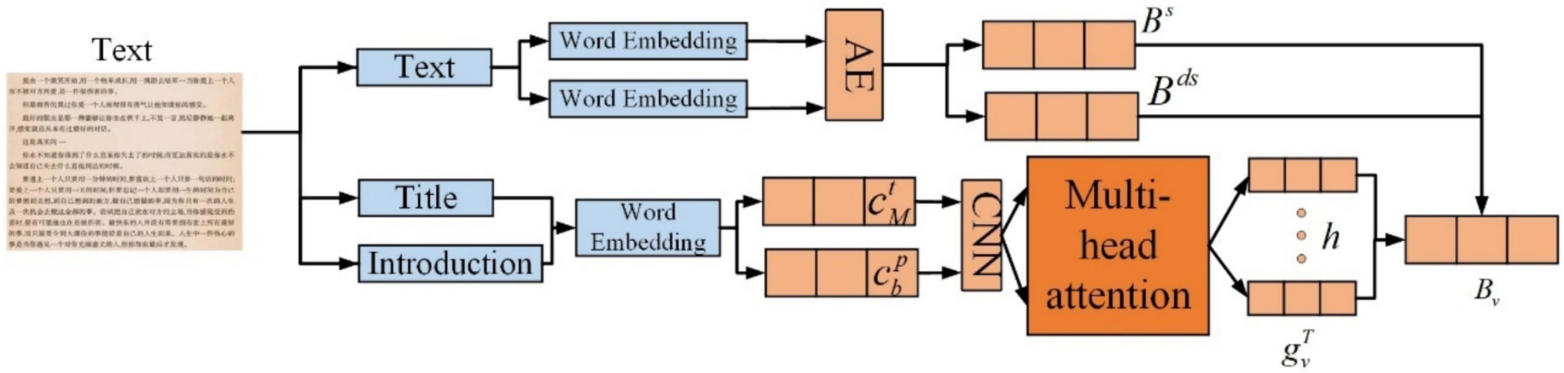
Structure of text feature extraction.

Structure of text feature extraction is presented in Figure 6. Lastly, since user preferences may be related and users tend to browse items with similar categories, a multi-head self-attention mechanism is used to capture interactions between similar items, enhancing the representation of the user. The $k_{\mathrm{th}}$ attention head’s representation of the $i_{\mathrm{th}}$ item is calculated as presented in Equation 25:

(25) $$\begin{array}{c} \beta_{i,j}^{k}=\frac{\exp(B_{i}^{T}Q_{k}^{n}B_{j})}{\sum_{m=1}^{M}\exp(B_{i}^{T}Q_{k}^{n}B_{m})}\\ h_{i,j}^{n}=V_{k}^{n}(\sum_{j=1}^{M}\beta_{i,j}^{k}B_{j}) \end{array}$$

where $Q_{k}^{n}$ and $V_{k}^{n}$ are the self-attention head parameters, and $\beta_{i,j}^{k}$ represents the similarity between the $j_{\mathrm{th}}$ and $i_{\mathrm{th}}$ items. The multi-head representation for the $i_{\mathrm{th}}$ item is the concatenation of the representations from the *h* independent attention heads.

**Figure 7:**
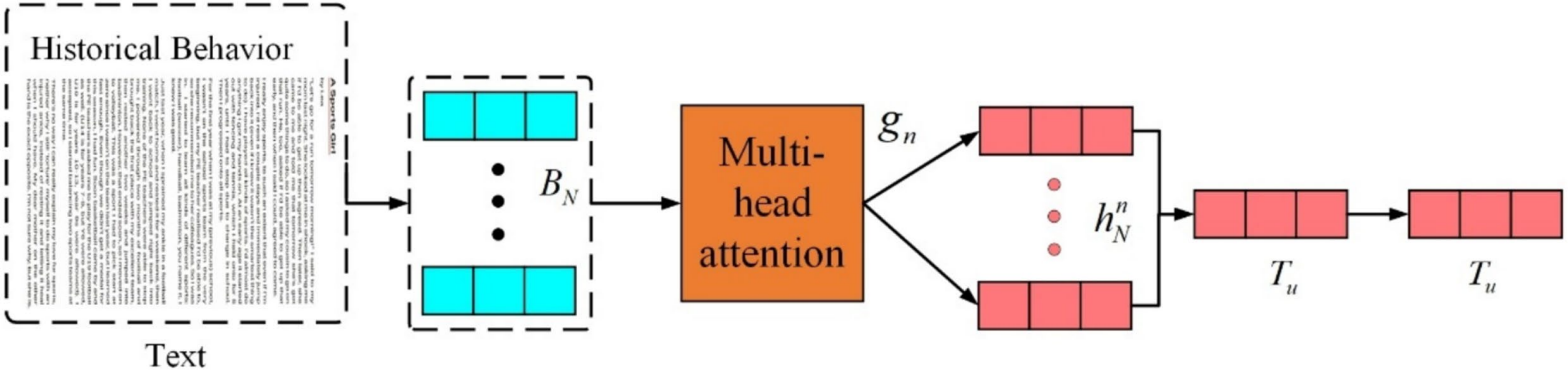
Structure of user feature extraction.

The user feature extraction part is shown in Figure 7. For user representation, the amount of user feature information carried by different items varies. Therefore, an attention mechanism is employed to better learn the user representation. The attention weight for the $i_{\mathrm{th}}$ item is calculated as presented in Equation 26:

(26) $$\begin{array}{c} a_{i}^{n}=q_{n}^{T}\tanh(V_{n}\times h_{i}^{h}+v_{n})\\ a_{i}^{n}=\frac{\exp(a_{i}^{n})}{\sum_{j=1}^{N}\exp(a_{J}^{n})} \end{array}$$

### 3.4 Prediction layer

After processing through the collaborative knowledge graph neural layer, the image feature extraction layer, and the text feature extraction layer, user and item features are obtained. Let 𝑄_𝑢_ and 𝑄_𝑣_ represent the sets of user and item features, respectively. The calculation process is presented in Equation 27:

(27) $$\begin{array}{c} Q_{u}=\{u_{G},u_{p},T_{u}\}\\ Q_{v}=\{v_{o},p_{v},B\} \end{array}$$

These feature vectors are concatenated to form the final vector representation of the user and item, as expressed by Equation 28:

(28) $$\begin{array}{c} e_{u}=u_{G}\;\|u_{p}\|\;T_{u}\\ e_{v}=v_{o}\;\|p_{v}\|\;B \end{array}$$

The final step involves applying the dot product of the user and item vectors to estimate the target user’s preference score for a specific item. The calculation is presented in Equation 29:

(29) $${\hat{y}}_{\mathrm{uv}}=e_{u}^{T}e_{v}$$

## 4 Experimental results and analysis

### 4.1 Dataset description

Two publicly available cross-domain recommendation datasets were employed in this study, covering the domains of movies and books: *MovieLens-1M* and *Amazon-Book*. The MovieLens-1M dataset, provided by the GroupLens research group, has been widely used in movie recommendation research and contains user rating records for movies (Harper and Konstan, 2015). The Amazon-Book dataset, extracted from the book subset of the Amazon Review Corpus, captures user rating behaviors toward book products (He and McAuley, 2016). To ensure experimental consistency, interaction records in each dataset were partitioned into a training set (80%), a test set (10%), and a validation set (10%, sampled from the training set) for model tuning. Table 1 summarizes the key statistics of the two datasets, including the number of users, items, and interactions, as well as data sparsity. Additionally, it presents the corresponding KG statistics, including the number of entities, relations, and total triples.

**Table 1:** Statistics of the datasets and corresponding knowledge graphs.

Dataset	User-project interaction				Knowledge graph related		
	Users	Items	Interactions	Sparsity	Entities	Relations	Triples
Movielens-1M	6,040	3,623	836,478	96.18%	73,988	51	385,923
Amazon-Book	70,679	24,915	847,733	99.95%	88,572	39	2,557,746

From Table 1, it is observed that the Movielens-1M dataset has 836,478 interactions and a sparsity of 96.18%, with 385,923 triples in its knowledge graph. The Amazon-Book dataset contains 8,477,733 interactions, with a sparsity of 99.95% and 2,557,746 triples in its knowledge graph.

### 4.2 Performance metrics

The evaluation metrics used in this experiment are Recall and NDCG, both of which accurately describe recommendation performance.

#### 4.2.1 Recall

This metric measures how many relevant items are correctly predicted within the top-X recommendations. It is computed as Equation 30:

(30) $$\text{Recall}=\frac{\mathrm{TP}}{\left(\mathrm{TP}+\mathrm{FN}\right)}$$

where *TP* represents the number of true positives, and *FN* represents the number of false negatives.

#### 4.2.2 NDCG

NDCG gives more weight to higher-ranked results, reflecting the diminishing relevance of items further down the ranking. It is defined as Equation 31:

(31) $$\text{NDCG}=\frac{\mathrm{DCG}}{\text{iDCG}}$$

where 𝐷𝐶𝐺 represents the discounted cumulative gain, and the weights of the arrangement order are summed. The earlier it is, the greater the proportion; 𝐷𝐶𝐺 is the best arranged 𝐷𝐶𝐺.

### 4.3 Baseline models and experimental setup

The following five baseline models were used in the experiments:

CKE (Zhang et al., 2016): A model based on collaborative filtering, which integrates text, image, and structural features within a single framework and uses TransR to enhance matrix factorization.
RippleNet (Wang et al., 2018): A model that continuously explores user preferences by incorporating a knowledge graph into the recommendation system, mitigating the cold-start problem.
KGNN-LS (Wang H. W. et al., 2019): This model constructs personalized graph representations for users using knowledge graphs, considering users’ unique preferences for different relations within the knowledge graph. It introduces label smoothing to improve generalization.
KGAT (Wang X. et al., 2019): A model that uses attention mechanisms to aggregate higher-order information between entities, addressing sparsity issues and improving recommendation accuracy.
KGECF (Zhang et al., 2021): A knowledge graph-based recommendation system that extracts latent features related to items, creating a personalized knowledge subgraph. It employs an end-to-end collaborative learning framework to merge knowledge graph and user behavior data for higher accuracy.

In light of current trends in KG-based recommendation research, although several emerging models have been proposed, the aforementioned five baseline methods remain representative in key aspects such as multimodal fusion, graph-based modeling, and user preference construction. These methods have also been extensively validated across various datasets, providing a solid foundation for comparative analysis. To further enhance readability, Table 2 summarizes the modalities utilized and feature fusion strategies adopted by each model. This comparison highlights the superiority of the proposed method in terms of its multimodal fusion capabilities.

**Table 2:** Comparison of modality usage and feature fusion mechanisms across different recommendation models.

Model	Collaborative filtering	Knowledge graph	Graph modeling	Image modality	Text modality	Modality fusion mechanism
CKE (Zhang et al., 2016)	✔	✔	✘	✔	✔	Multimodal joint matrix factorization + TransR
RippleNet (Wang et al., 2018)	✔	✔	✘	✘	✘	KG path propagation mechanism
KGNN-LS (Wang H. W. et al., 2019)	✔	✔	✔	✘	✘	Label smoothing-based GCN with personalized aggregation
KGAT (Wang X. et al., 2019)	✔	✔	✔ (GAT)	✘	✘	Graph attention mechanism for high-order relations
KGECF (Zhang et al., 2021)	✔	✔	✔	✘	✘	Subgraph generation + joint collaborative modeling
Ours	✔	✔	✔ (HGNN + GRU)	✔	✔	Graph attention + GRU + multi-path visual attention + multi-head text attention

As shown in the table, the proposed model not only maintains strong collaborative modeling capabilities but also systematically integrates knowledge graph, image, and text modalities. By incorporating a more fine-grained multi-path attention mechanism and a hierarchical structure, the model enables a more comprehensive representation of user preferences, thereby enhancing both recommendation accuracy and generalization performance.

The experiments were conducted on a Microsoft Windows 10 system, with an Nvidia 3,070 GPU, 32 GB of memory, and an AMD R7-6800H processor. The programming language used is Python 3.8.6, and PyTorch is utilized to implement the models. Item and user embedding sizes were set to 64, with a batch size of 256. The Adam optimizer is used with a learning rate of 0.002, and model parameters were initialized using the Glorot method. The training, validation, and test sets were split in an 80:10:10 ratio based on recommendation evaluation metrics.

### 4.4 Comparison of model performance

In this experiment, the proposed model is compared with the four baseline models across two datasets. Since recommendation accuracy is influenced by the number of recommendations, X is set to {5, 10, 15, 20, 25}. The performance results on the Movielens and Amazon-Book datasets are shown in Tables 3, 4, respectively.

**Table 3:** Performance comparison on Movielens-1M dataset.

Model	Movielens-1M				
	Recall				
	5	10	15	20	25
CKE	0.0506 ± 0.0012	0.0627 ± 0.0011	0.0755 ± 0.0013	0.0805 ± 0.0016	0.0953 ± 0.0016
RippleNet	0.0479 ± 0.0010	0.0650 ± 0.0013	0.0801 ± 0.0014	0.0840 ± 0.0013	0.1033 ± 0.0015
KGNN-LS	0.1165 ± 0.0023	0.1521 ± 0.0018	0.1868 ± 0.0025	0.2251 ± 0.0027	0.2626 ± 0.0028
KGAT	0.1235 ± 0.0020	0.1647 ± 0.0024	0.2005 ± 0.0024	0.2417 ± 0.0026	0.2714 ± 0.0035
KGECF	0.1364 ± 0.0019	0.1771 ± 0.0021	0.2285 ± 0.0031	0.2669 ± 0.0027	0.2980 ± 0.0038
Ours	0.1440 ± 0.0017	0.1869 ± 0.0022	0.2337 ± 0.0028	0.2772 ± 0.0024	0.3034 ± 0.0031

**Table 4:** Performance comparison on Amazon-Book dataset.

Model	Amazon-Book				
	Recall				
	5	10	15	20	25
CKE	0.0438 ± 0.0010	0.0691 ± 0.0012	0.0737 ± 0.0012	0.0820 ± 0.0013	0.0888 ± 0.0013
RippleNet	0.0480 ± 0.0012	0.0674 ± 0.0015	0.0752 ± 0.0011	0.0841 ± 0.0013	0.0941 ± 0.0011
KGNN-LS	0.0852 ± 0.0014	0.1183 ± 0.0017	0.1244 ± 0.0015	0.1566 ± 0.0022	0.1775 ± 0.0024
KGAT	0.0952 ± 0.0016	0.1288 ± 0.0016	0.1389 ± 0.0018	0.1661 ± 0.0027	0.1804 ± 0.0029
KGECF	0.1068 ± 0.0016	0.1340 ± 0.0020	0.1507 ± 0.0022	0.1771 ± 0.0029	0.1849 ± 0.0026
Ours	0.1133 ± 0.0014	0.1415 ± 0.0017	0.1583 ± 0.0019	0.1850 ± 0.0027	0.1953 ± 0.0026

The baseline comparison analysis for the two datasets shows that the recall rate (Recall) of all models increases with the number of recommendations (X). In this trend, the MKGAR model proposed in this study demonstrates the highest recall rate, significantly outperforming other models. This result confirms that the MKGAR model substantially improves the performance of recommendation systems.

Upon analyzing the experimental results, it is observed that the CKE and RippleNet models performed relatively poorly, while KGNN-LS, KGAT, and KGECF performed better. Specifically, the CKE model did not incorporate the TransR method, which led to an inability to fully capture the complex structure of the knowledge graph. This confirms the superiority of the TransR method in handling knowledge graph information. In contrast, RippleNet showed slight improvements over CKE by utilizing a knowledge graph, but it failed to fully explore higher-order connectivity and collaborative signals from users.

For the Recall metric, CKE and RippleNet exhibited similar performance across both datasets, while the proposed model showed a significant improvement over both. On the other hand, KGNN-LS, KGAT, and KGECF demonstrated clear advantages. KGNN-LS, which combines knowledge graphs and label smoothing regularization, improves recommendation accuracy and interpretability but focuses primarily on learning local structural features, potentially overlooking the impact of global information. The KGAT model, despite aggregating information by calculating spatial relationships between head and tail entities, did not adequately account for interactions between users. The KGECF model utilizes an attention mechanism to efficiently extract higher-order relational information from the knowledge graph, and integrates users’ long-term and short-term preferences through gated neural networks, significantly improving recommendation accuracy. However, these models do not account for the potential influence of text and image features on the recommendations, resulting in lower performance compared to the proposed model. The proposed model leverages multi-perspective features—particularly textual and visual modalities—which play a crucial role in enhancing recommendation accuracy. This effectiveness has been empirically validated. For instance, in terms of Recall, the proposed model demonstrates significant improvements over KGNN-LS, KGAT, and KGECF on both the MovieLens-1M and Amazon-Book datasets. These results indicate that by effectively mining latent information from multiple modalities, the proposed approach exhibits strong recommendation capabilities.

Based on the results of hypothesis testing (two-sample *t*-test, *n* = 5, two-tailed test assuming equal variances), the *p*-values for the proposed method compared with each baseline across different recall positions on the two datasets are presented in Tables 5, 6. Significance levels are denoted as follows: *p* < 0.05 (*), *p* < 0.01 (**). The detailed results of the significance tests are shown below.

**Table 5:** *p*-values on the MovieLens-1M dataset.

Model	Movielens-1M				
	Recall@5	Recall@10	Recall@15	Recall@20	Recall@25
CKE	0.0000**	0.0000**	0.0000**	0.0000**	0.0000**
RippleNet	0.0000**	0.0000**	0.0000**	0.0000**	0.0000**
KGNN-LS	0.0000**	0.0000**	0.0000**	0.0000**	0.0000**
KGAT	0.0000**	0.0000**	0.0000**	0.0000**	0.0000**
KGECF	0.0003**	0.0000**	0.0238*	0.0011**	0.3232

**Table 6:** *p*-values on the Amazon-Book dataset.

Model	Amazon-Book				
	Recall@5	Recall@10	Recall@15	Recall@20	Recall@25
CKE	0.00000**	0.00000**	0.00000**	0.00000**	0.00000**
RippleNet	0.00000**	0.00000**	0.00000**	0.00000**	0.00000**
KGNN-LS	0.00000**	0.00000**	0.00000**	0.00000**	0.00001**
KGAT	0.00000**	0.00001**	0.00000**	0.00000**	0.00004**
KGECF	0.00013**	0.00021**	0.00038**	0.00212*	0.00023**

As observed from Tables 5, 6, the proposed method demonstrates statistically significant improvements over all baseline models under most settings, with *p* < 0.01 in the majority of comparisons. In particular, the comparison with KGECF at Recall@15 on MovieLens-1M and Recall@20 on Amazon-Book yields *p*-values slightly above the 0.01 threshold but still below 0.05, indicating moderate significance. The only non-significant result occurs at Recall@25 on MovieLens-1M when compared with KGECF (*p* = 0.3232). These significance testing results further verify the robustness and consistent performance improvements of the proposed model across different recall levels and datasets.

### 4.5 Ablation study

This section explores how user preferences related to visual and semantic features, derived from item images and text, can be integrated into item embeddings. To assess the specific impact of image and text features on the performance of the recommendation system, experiments were designed by modifying the user and item feature components in Equation 28. The models were then evaluated by removing either the image features (model labeled HGAN-MKG1) or the text features (model labeled HGAN-MKG2). By comparing these ablated models with the full model (MKGAR) across different top-*K* values (5, 10, 20, 25), we can gain detailed insights into the contribution of each modality to the recommendation results. The detailed metrics including NDCG and Recall are reported in Table 7.

**Table 7:** Comparative experimental results of ablation study.

Top-K	Model	Movielens-1M (NDCG/Recall)	Amazon-Book (NDCG/Recall)
5	HGAN-MKG1	0.1771/0.1427	0.0581/0.0628
	HGAN-MKG2	0.1756/0.1425	0.0578/0.0623
	MKGAR	0.1783/0.1440	0.0594/0.1133
10	HGAN-MKG1	0.2202/0.1846	0.0753/0.1395
	HGAN-MKG2	0.2217/0.1841	0.0753/0.1402
	MKGAR	0.2240/0.1869	0.0774/0.1415
15	HGAN-MKG1	0.2858/0.2330	0.0844/0.1568
	HGAN-MKG2	0.2840/0.2321	0.0846/0.1573
	MKGAR	0.2875/0.2337	0.0869/0.1583
20	HGAN-MKG1	0.3343/0.2746	0.1045/0.1840
	HGAN-MKG2	0.3348/0.2754	0.1037/0.1836
	MKGAR	0.3361/0.2772	0.1064/0.1850
25	HGAN-MKG1	0.3759/0.3005	0.1086/0.1939
	HGAN-MKG2	0.3762/0.3009	0.1081/0.1935
	MKGAR	0.3777/0.3034	0.1114/0.1953

As seen from the above results, both image and text features contribute positively to model performance across all top-*K* values. However, the magnitude of their impact is relatively modest. This is attributed to the redundancy and strong expressive power of the knowledge graph-based structural features, which may already encode rich item semantics and relationships. Thus, the additional visual and textual modalities, though helpful, offer only incremental improvements. These results highlight the robustness of the knowledge-aware representation while also confirming that multi-modal auxiliary features can enhance performance, especially under sparse or cold-start conditions.

### 4.6 Hyperparameter experiment

**Figure 8:**
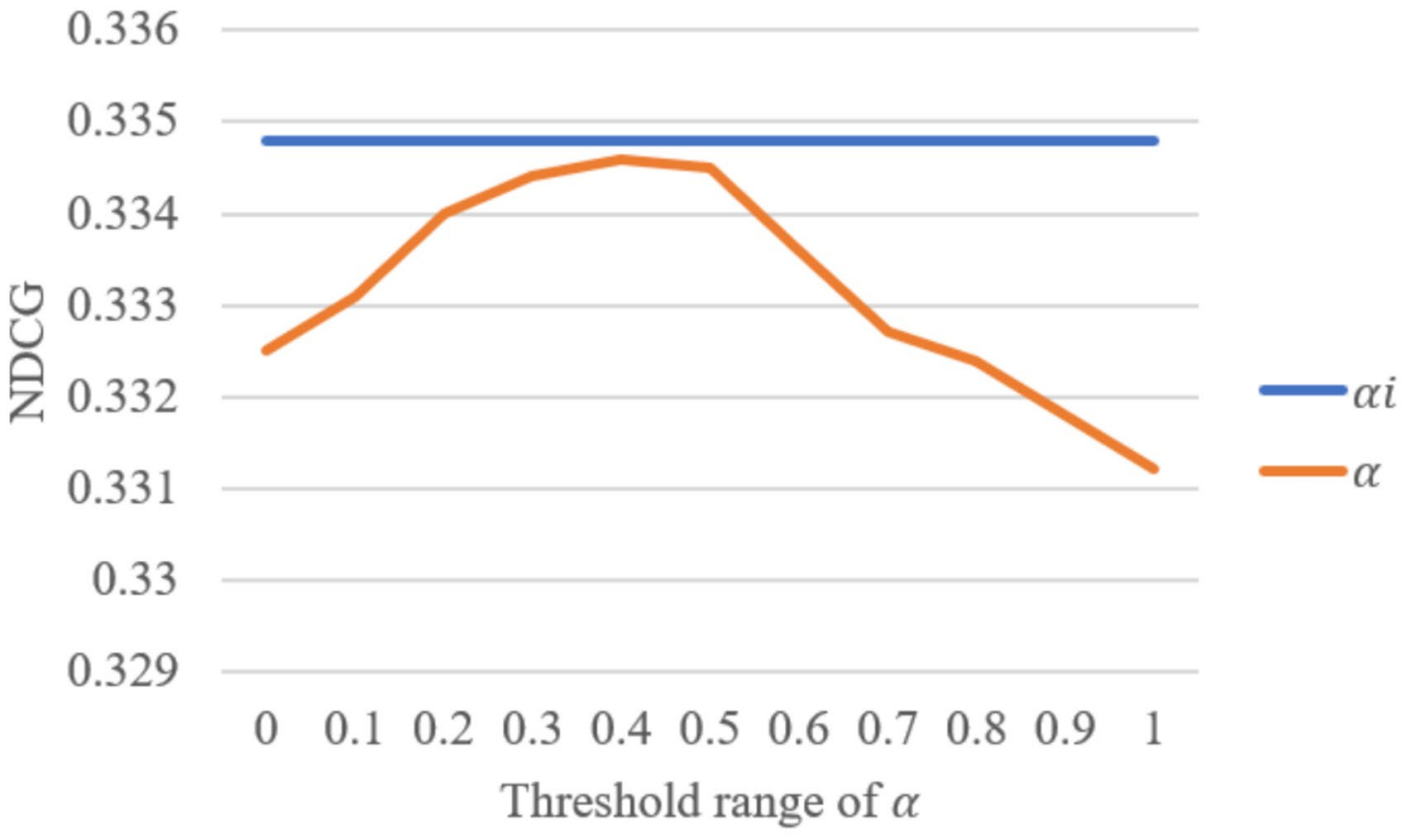
Impact of the attention coefficient *α* in the knowledge graph attention network layer on model performance.

In the collaborative knowledge graph neural layer, the attention coefficient 𝛼_𝑖_ plays a crucial role, as the attention score depends on the spatial relationship between entities in the knowledge graph. To evaluate the impact of the attention coefficient on the model performance, we varied the value of 𝛼 within the range [0, 1] and compared the results with those obtained from the original model using 𝛼_𝑖_. The performance is evaluated using NDCG on the Amazon-Book dataset with the number of recommendations set to 20. The changes in NDCG with different values of *α*\alphaα are shown in Figure 8, illustrating the impact of different attention coefficients on model performance.

The analysis of the data in Figure 8 shows that when the attention coefficient 𝛼 is set to the value of 𝛼_𝑖_, the NDCG reaches its peak at 0.3348. No value of 𝛼 within the range [0, 1] exceeded this performance. Specifically, as 𝛼 increases, the performance initially rises to a peak and then begins to decline. The highest NDCG is observed at 𝛼=0.4 with a value of 0.3346, while at 𝛼=1, the performance dropped to 0.3312. Notably, when 𝛼=1, the performance improved by 1.09% compared to the original 𝛼_𝑖_ value. This result indicates that dynamically adjusting the attention coefficient based on the spatial relationship between entities, rather than using a static threshold, can more effectively enhance model performance. This is because a smaller threshold may introduce noise from irrelevant entities, while a higher threshold may excessively filter out nodes, reducing the amount of information. Therefore, dynamically adjusting the attention coefficient to align with the spatial relationships of entities is an effective strategy for optimizing recommendation system performance.

## 5 Conclusion

This paper introduces a recommendation method that combines hierarchical graph attention networks and multimodal knowledge graphs. The core components of the approach include the integration of knowledge graphs with graph neural attention mechanisms and multimodal feature fusion. In the collaborative knowledge graph neural layer, the knowledge graph serves as a structured representation, encompassing a wealth of entities, relationships, and attributes, while graph neural networks help uncover deeper collaborative relationships. Additionally, by incorporating image and text features (such as movie posters, book covers, names, descriptions, and categories), the model enhances its understanding of users’ visual and semantic preferences. This approach not only effectively models user interests but also provides more accurate recommendations. Extensive experiments on the MovieLens and Amazon-Book datasets demonstrate that the proposed model significantly outperforms other knowledge graph-based recommendation models in terms of performance.

## Data Availability

The datasets presented in this study can be found in online repositories. The names of the repository/repositories and accession number(s) can be found at: https://grouplens.org/datasets/movielens/.
